# Activation of Akt pathway by transcription-independent mechanisms of retinoic acid promotes survival and invasion in lung cancer cells

**DOI:** 10.1186/1476-4598-12-44

**Published:** 2013-05-21

**Authors:** Alejandro García-Regalado, Miguel Vargas, Alejandro García-Carrancá, Elena Aréchaga-Ocampo, Claudia Haydée González-De la Rosa

**Affiliations:** 1 Departamento de Ciencias Naturales, Universidad Autónoma Metropolitana, Unidad Cuajimalpa. Artificios 40, Col. Hidalgo, México, D. F 01120, Mexico; 2 Departamento de Biomedicina Molecular, Centro de Investigación y de Estudios Avanzados del IPN, Av. Instituto Politécnico Nacional 2508, Col. San Pedro Zacatenco, México, D. F 14740, Mexico; 3 Subdirección de Investigación Básica, Instituto Nacional de Cancerología, San Fernando 22, Col Sección XVI, México, D. F 14080, Mexico

**Keywords:** Lung cancer, ATRA, Resistance, PI3k/Akt pathway, RARs, RAC, Cell invasion, Apoptosis

## Abstract

**Background:**

All-*trans* retinoic acid (ATRA) is currently being used in clinical trials for cancer treatment. The use of ATRA is limited because some cancers, such as lung cancer, show resistance to treatment. However, little is known about the molecular mechanisms that regulate resistance to ATRA treatment. Akt is a kinase that plays a key role in cell survival and cell invasion. Akt is often activated in lung cancer, suggesting its participation in resistance to chemotherapy. In this study, we explored the hypothesis that activation of the Akt pathway promotes resistance to ATRA treatment at the inhibition of cell survival and invasion in lung cancer. We aimed to provide guidelines for the proper use of ATRA in clinical trials and to elucidate basic biological mechanisms of resistance.

**Results:**

We performed experiments using the A549 human lung adenocarcinoma cell line. We found that ATRA treatment promotes PI3k-Akt pathway activation through transcription-independent mechanisms. Interestingly, ATRA treatment induces the translocation of RARα to the plasma membrane, where it colocalizes with Akt. Immunoprecipitation assays showed that ATRA promotes Akt activation mediated by RARα-Akt interaction. Activation of the PI3k-Akt pathway by ATRA promotes invasion through Rac-GTPase, whereas pretreatment with 15e (PI3k inhibitor) or over-expression of the inactive form of Akt blocks ATRA-induced invasion. We also found that treatment with ATRA induces cell survival, which is inhibited by 15e or over-expression of an inactive form of Akt, through a subsequent increase in the levels of the active form of caspase-3. Finally, we showed that over-expression of the active form of Akt significantly decreases expression levels of the tumor suppressors RARβ2 and p53. In contrast, over-expression of the inactive form of Akt restores RARβ2 expression in cells treated with ATRA, indicating that activation of the PI3k-Akt pathway inhibits the expression of ATRA target genes.

**Conclusion:**

Our results demonstrate that rapid activation of Akt blocks transcription-dependent mechanism of ATRA, promotes invasion and cell survival and confers resistance to retinoic acid treatment in lung cancer cells. These findings provide an incentive for the design and clinical testing of treatment regimens that combine ATRA and PI3k inhibitors for lung cancer treatment.

## Background

Lung cancer is the leading cause of deaths due to cancer worldwide [1]. Sixty percent of cases are diagnosed in advanced stages, with a life expectancy of less than one year [2]. Chemotherapy treatment is typically administered in these stages; however, the response rate is only about 9% [3]. Clinical trials have shown potential for chemical compounds in cancer treatment such as all-*trans* retinoic acid (ATRA), which shows anti-proliferative and apoptotic effects and a role in modulating cellular invasion [4]. ATRA exerts its cellular effects by inducing changes in gene expression and is now also thought to be a rapid modulator of signaling pathways involved in cancer. However, the mechanisms mediating these rapid effects are not yet well understood.

ATRA is a biologically active metabolite of vitamin A that regulates diverse cellular functions such as differentiation, proliferation and apoptosis [5-7]. The functions of ATRA are mediated by nuclear receptors, specifically the retinoic acid receptors (RAR α, β, and γ) and the retinoic X receptors (RXR α, β, and γ). RARs act as retinoid-inducible transcriptional factors and can form heterodimers with RXRs, which regulate the expression of genes involved in cell cycle arrest, cell differentiation and cell death [8]. The RARβ2 gene is one of the genes whose expression increases upon ATRA treatment. RARβ2 is a tumor suppressor whose expression is regulated by RARα in response to ATRA [9] and several reports indicate that the expression of RARβ2 is significantly decreased in human cancers [10].

Recent studies have demonstrated that ATRA induces rapid, transcription-independent activation of the PI3k/Akt pathway in neuroblastoma cells [11]. However, the molecular mechanisms by which ATRA promotes activation of the PI3k/Akt pathway are still unknown. The PI3k/Akt pathway is deregulated in most human cancers, including non-small cell lung cancer (NSCLC) [12-14]. Phosphoinositide 3-kinase (PI3k) is activated by stimulation of multiple receptor tyrosine kinases and G protein-coupled receptors. Active PI3k catalyzes the production of phosphatidylinositol-3,4,5-triphosphate (PIP(3)) at the plasma membrane, which in turn promotes the recruitment and activation of Akt at the membrane [15]. Akt is a serine/threonine kinase that plays a key role in multiple cellular processes, such as proliferation, survival and cell invasion [16]. Over-activation of Akt influences multiple downstream effectors, including inactivation of proapoptotic factors such as Bad and caspase-9 [17,18].

ATRA is currently being used in clinical trials for lung cancer treatment; however, its use is limited because lung cancers show resistance to treatment with ATRA [19-22]. Little is known about the molecular mechanisms that regulate resistance to ATRA treatment in lung cancer. In this report, we tested the hypothesis that Akt mediates resistance to ATRA treatment by treating A549 cells with ATRA and assessed the functional relevance of Akt inactivation in apoptosis and invasion. The A549 cell line is highly invasive, metastatic and resistant to proliferative and survival inhibitory effects of ATRA [23-25].

## Results

### ATRA promotes activation of the PI3k/Akt pathway by inducing the association of RARα with Akt via transcription-independent mechanisms

**Figure 1:**
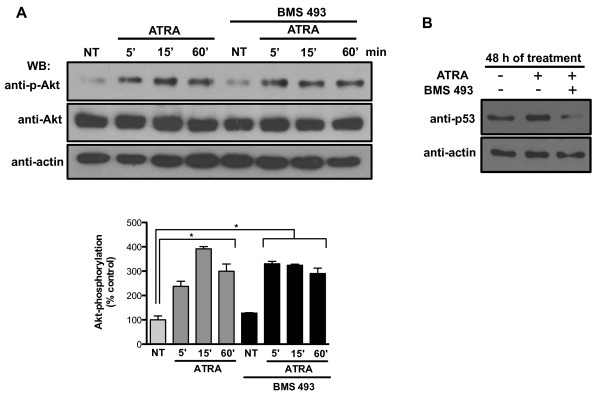
**ATRA activates the Akt pathway through non-genomic mechanisms in A549 cells*. ***(**A**) Left, A549 cells were serum-starved for 18 h, treated or non-treated (NT) with 5 μM of ATRA, for the times indicated. Right, A549 cells were preincubated for 1 h with 3 μM of BMS493 before ATRA treatment and total extracts were prepared. The phosphorylated form of Akt and total proteins were detected by western blot using specific antibodies. The bottom graph represents the densitometric values of Akt phosphorylation of three independent experiments (means ± SEM, *P < 0.05; **P < 0.001 compared with non-treated cells (NT) (analysis of variance and Newman-Keuls test). (**B**) A549 cells were serum-starved and treated with ATRA with or without BMS493 for 48 h. Total proteins were detected by western blot.

To investigate the molecular mechanisms of ATRA resistance in lung cancer cells, we investigated the effects of ATRA in regulating the PI3k/Akt pathway in the ATRA-resistant A549 cell line [26,27]. The results revealed a rapid activation of the PI3k/Akt pathway, measured by Akt phosphorylation at its serine 473, within 5 min of ATRA treatment and until 60 min after treatment (Figure 1A). Similar results were obtained for H1944, another lung adenocarcinoma cell line, whereas in NL-20, a normal lung cell line, Akt phosphorylation was only detected at 15 min of treatment (Additional file 1: Figure S1). To examine the transcription-dependent action of ATRA on Akt activation, we used BMS493, a pan-retinoic acid receptor antagonist (Figure 1A). Interestingly, treatment with BMS493 did not prevent Akt activation. The effectiveness of BMS493 treatment was evaluated by testing its ability to counteract the transcription-dependent effect of ATRA on p53 expression [28]. As expected, BMS493 inhibited the ATRA-induced increase in p53 expression levels (Figure 1B).

**Figure 2:**
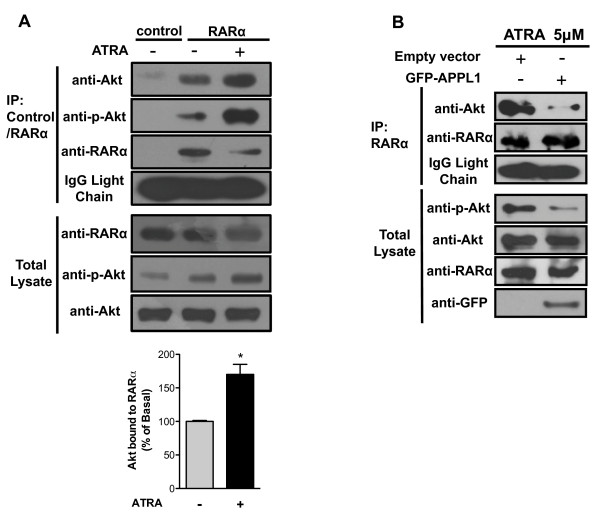
**ATRA promotes Akt activation mediated by RARα-Akt interaction.** (**A**) RARα was immunoprecipitated from A549 cells treated or non-treated with 5 μM of ATRA for 15 min. Immunoprecipitated RARα and associated protein were detected by western blot. Control refers to immunoprecipitation performed with an Erk1 antibody. The bottom graph shows the results of densitometric analyses of Akt bound to RARα obtained from three independent experiments (means ± SEM, *P < 0.05 compared with non-treated cells (NT) assessed by *t* test analysis). (**B**) RARα was immunoprecipitated from A549 cells transfected with EGFP-APPL1 or empty vector and treated with 5 μM of ATRA for 15 min. Association of RARα with Akt was detected by western blot using specific antibodies. Image shows one representative experiment of three independent.

Since ATRA promotes Akt activation, we decided to test whether Akt interacts with components of ATRA signaling. RARα is a major mediator of non-genomic ATRA effects and is widely expressed in all tissue types [29,30]. To determine whether Akt interacts with RARα, we immunoprecipitated RARα from non-treated or ATRA treated cells. As show in Figure 2A and B, ATRA treatment promoted a significant increase in the interaction between Akt and RARα, with RARα showing a higher binding affinity to the phosphorylated form of Akt. We next determined whether the activation of Akt depends on its interaction with RARα. For this, we tested whether the interaction between RARα and Akt could be competed with APPL1, a protein that interacts directly with Akt [31-33]. Figure 2B shows that over-expression of APPL1 blocks the interaction between RARα with Akt, and inhibits ATRA-mediated Akt activation.

### ATRA stimulates the translocation of RARα to the plasma membrane, activates Rac and increases membrane ruffles

**Figure 3:**
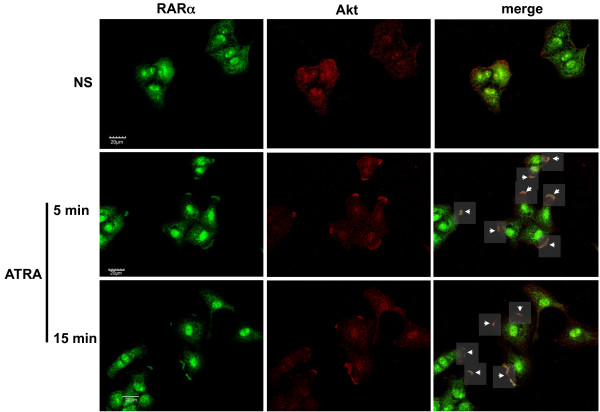
**ATRA promotes recruitment of RARα to the plasma membrane*. ***A549 cells were serum-starved and treated with 5 μM of ATRA for the times indicated. Then cells were fixed and incubated with anti-RARα and anti-Akt followed by incubation with anti-mouse Alexa Fluor 532 and Alexa Fluor 647, respectively, as described in *Materials and Methods.* Finally, the cells were analyzed by confocal microscopy.

To determine the influence of ATRA on the subcellular distribution of RARα and Akt, A549 cells were treated with ATRA for different amounts of time and localization of these proteins was examined by immunofluorescence (Figure 3). In non-treated cells, RARα was predominantly found in the nucleus and Akt was located in the plasma membrane and cytoplasm. In contrast, cells treated with ATRA showed RARα recruitment to the plasma membrane from the 5th min to the 15th min of treatment and RARα was co-localized with Akt in newly formed ruffles (white arrows in Figure 3).

**Figure 4:**
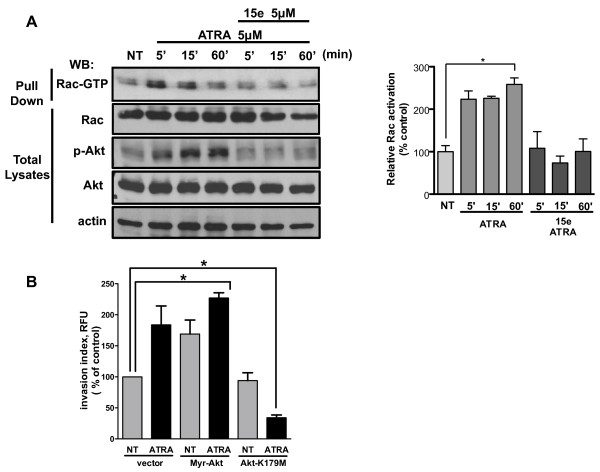
**ATRA stimulates Rac activation and promotes invasion.** (**A**) Left, A549 cells were serum-starved for 18 h and treated with 5 μM of ATRA for the times indicated. Other cells were preincubated for 1 h with 5 μM of 15e. Activated Rac was detected with the Rac1 Activation assay kit according to the manufacturer’s instructions. Right, the graph shows the results of densitometric analysis of relative increase of Rac activation obtained in three independent experiments. (**B**) Cell invasion was analyzed by QCM™ 24–well Invasion Assay Kit. A549 cells were transfected with Myr-Akt, Akt-K179M or empty vector and seeded at 2.5 × 10^5^ cells/well into the upper chamber. DMEM/F12 was added to the lower chamber with or without 5 μM ATRA for 48 h. The invasive cells were detected according to the manufacturer’s instructions. The graphs shows the results of three independent experiments (means ± SEM, *P < 0.05 compared with non-treated cells (NT) (analysis of variance and Newman-Keuls test).

Activation of Rac-GTPase is a critical step leading to membrane protrusion and ruffle formation [34,35]. To assess whether ATRA stimulates Rac activation, we evaluated the interaction of recombinant PAK (p21-activated kinase) with GTP-Rac by pull-down. As shown in Figure 4A, the amount of GTP-bound Rac increased in a time-dependent manner in cells treated with ATRA, whereas the pretreatment of cells for 1 h with PI3k inhibitor (15e) prevented Rac activation.

### ATRA promotes cell invasion

The Akt signaling pathway has been previously implicated in cell invasion. To determine the functional consequences of Akt activation by ATRA, we transiently transfected A549 cells with a constitutively active form of Akt (Myr-Akt) and an inactive form of Akt (K179M) and evaluated invasion. As shown in Figure 4B, ATRA promoted invasion in cells expressing empty vector and over-expression of Myr-Akt increased invasion in cells regardless of treatment with ATRA. However, over-expression of Akt-K179M blocked the effect of ATRA on invasion.

### Inhibition of the PI3k/Akt pathway blocks the ATRA-dependent survival effect by activating caspase-3

**Figure 5:**
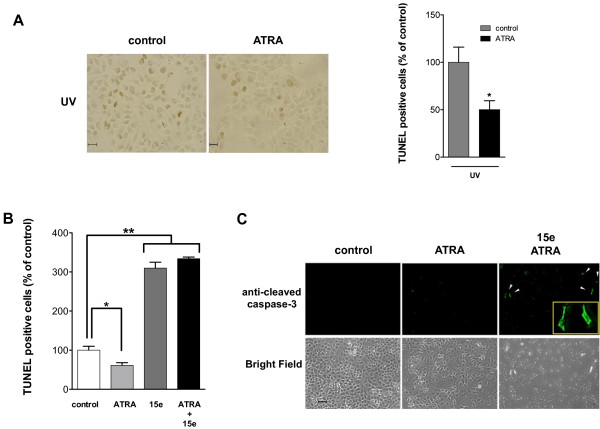
**Inhibition of the PI3k/Akt pathway promotes apoptosis by activation of caspase-3. **(**A**) Left, A549 cells were serum-starved and treated or non-treated (control) with ATRA for 48 h, during the first 12 h after treatment with ATRA, the cells were irradiated with 150 J/m^2^ of UV-C light for 30 min. Subsequently, DNA fragmentation was detected by TUNEL according to the manufacturer’s instructions. The apoptotic cells are stained brown. Bar, 20 μm. Right, percentages of TUNEL-positive cells were quantified by counting 200 cells from four random microscopic fields (means ± SEM, *P < 0.05 compared with non-treated cells (control) assessed by *t* test analysis). (**B**) A549 cells were treated for 48 h with 5 μM of ATRA alone or combined with 5 μM of 15e. Subsequently, DNA fragmentation was detected by TUNEL. Control cells were non-treated. Percentages of TUNEL-positive cells were quantified by counting 200 cells from four random microscopic fields. Means ± SEM, *P < 0.05; **P < 0.001 compared with non-treated cells (control) (analysis of variance and Newman-Keuls test). (**C**) A549 cells were serum-starved and treated or non-treated (control) with 5 μM of ATRA alone or combined with 5 μM of 15e for 48 h. The cells were fixed, stained with anti-cleaved caspase-3 followed by donkey anti-goat FITC as described in *Materials and Methods* and analyzed by fluorescence microscopy. Bar, 20 μm.

We investigated the effects of ATRA on cell apoptosis by TUNEL assays. As shown in Figure 5A and B, ATRA protected A549 cells against apoptosis under stress conditions, such as ultraviolet (UV) radiation exposition and serum starvation, whereas treatment with PI3k inhibitor (15e) strongly promoted apoptosis (Figure 5B). The combined treatment with ATRA and 15e did not exert additive effects on apoptosis. To investigate the molecular mechanism of PI3k inhibitor-induced apoptosis in A549 cells, the expression of activated caspase-3 was determined by immunofluorescence microscopy. As shown in the bottom panel of Figure 5C, PI3k inhibitor (15e) treatment induced caspase-3 activation, whereas ATRA treatment alone did not affect caspase-3 activation.

**Figure 6:**
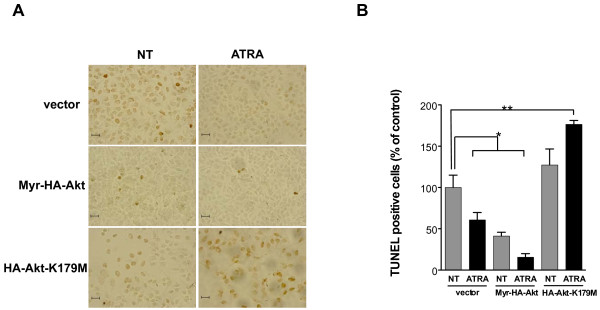
**Inactive form of Akt (K179M) blocks the ATRA-dependent survival effect. **(**A**) A549 cells were transfected with Myr-Akt, Akt-K179M or empty vector and subsequently treated or non-treated with 5 μM of ATRA for 48 h. Subsequently, DNA fragmentation was detected by TUNEL according to the manufacturer’s instructions. Control cells were non-treated. The apoptotic cells are stained brown. (**B**) Percentages of TUNEL-positive cells were quantified by counting 100 cells from three random microscopic fields. Means ± SEM, *P < 0.05; **P < 0.001 compared with non-treated cells (NT) (analysis of variance and Newman-Keuls test). Bar, 20 μm.

To investigate the direct effect of Akt on apoptosis in cells treated with ATRA, we transfected A549 cells with an active and inactive form of Akt. Figure 6 shows that over-expression of Myr-Akt increase the protective effects of ATRA on apoptosis, whereas over-expression of Akt-K179M promoted apoptosis in cells treated with ATRA. These results demonstrate that PI3k/Akt activation mediates the protective effect of ATRA on apoptosis.

### Activation of Akt blocks the ATRA-dependent transcription

**Figure 7:**
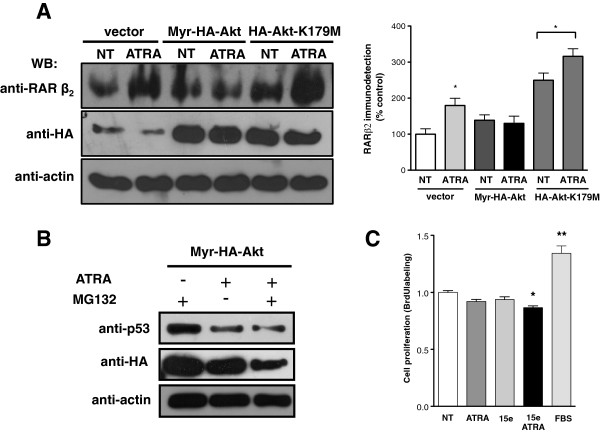
**Akt activation promotes the down-regulation of RARβ2 and p53. **(**A**) Left, A549 cells were transfected with Myr-Akt, Akt-K179M or empty vector and subsequently treated or non-treated with 5 μM of ATRA for 48 h. Total extracts were prepared and levels of protein were detected by western blot. Right, the graph shows the results of densitometric analysis of relative RARβ2 protein expression levels, obtained in three independent experiments (means ± SEM, *P < 0.05 compared with non-treated cells (NT) transfected with empty vector (analysis of variance and Newman-Keuls test). (**B**) A549 cells were transfected with Myr-Akt and subsequently treated or non-treated with 5 μM of ATRA for 48 h. For the last 24 h of the 48 h treatment period, the cells were incubated with 20 μM of MG132. Total extracts were prepared and levels of protein were detected by western blot using specific antibodies. The image shows one representative experiment of three independent. (**C**) A549 cells were serum-starved and treated or non-treated (control) with 5 μM of ATRA alone or in combination with 5 μM of 15e for 24 h. The proliferative effect was assessed by BrdU labeling according to the manufacturer’s instructions. The graph shows the results of three independent experiments (means ± SEM, *P < 0.05: **P < 0.001 compared with non-treated cells (NT) (analysis of variance and Newman-Keuls test).

To determine the effects of Akt on expression of target genes of ATRA such as RARβ2 and p53, we assessed the effect of ATRA in A549 cells transfected with an active and inactive form of Akt. Figure 7A shows that ATRA treatment significantly increased RARβ2 expression in cells transfected with the empty vector, whereas over-expression of Myr-Akt blocked ATRA-induced expression of RARβ2. However, over-expression of Akt-K179M enhanced the effect of ATRA on RARβ2 expression and similar results were obtained in cells treated with PI3k inhibitor (Additional file 2: Figure S2). Figure 7B shows that over-expression of Myr-Akt blocks the expression of p53 in cells treated with ATRA, whereas pretreatment with proteasome inhibitor (MG132) did not prevent Akt-induced decrease in p53 expression. Taken together, these results demonstrate that Akt activation promotes the down-regulation of RARβ2 and p53 at transcriptional level.

### Combined treatment of ATRA and PI3k inhibitor exerted a modest anti-proliferative effect

To examine the effect of ATRA on cell proliferation, A549 cells were treated for 24 h with ATRA or 15e. As shown in Figure 7C, neither ATRA nor 15e treatment affected proliferation when compared with the control (non-treated cells). Nevertheless, the combination of ATRA with 15e showed a modest anti-proliferative effect. Similar results were obtained when treatment was until 48 and 72 h (data not shown). These results suggest that the PI3k/Akt pathway partially regulates A549 cell proliferation.

## Discussion

ATRA is used in clinical trials to suppress the development of different types of cancer [26]. However, its effectiveness is limited in some cancers, such as lung cancer [20,21,36]. In this work, we demonstrate that resistance to ATRA-induced apoptosis and suppression of invasion of A549 lung cancer cells is mediated by activation of the PI3k/Akt pathway. Our results show that ATRA promotes phosphorylation of Akt through transcription-independent mechanisms. These data are consistent with reports showing that ATRA induces phosphorylation of Akt via transcription-independent mechanisms in neuroblastoma cells [11]. These results are supported by the use of pan-RAR antagonist (BMS493), which prevent expression of ATRA target genes, but not prevent Akt activation by ATRA. Such results suggest that the structural changes in retinoic acid receptors promoted by BMS493 increase its affinity for co-repressors in the nucleus, whereas in plasma membrane, these structural changes not prevent assembly of Akt-RAR complex. In agreement with this possibility, recent reports indicate that selective receptor modulators can display agonistic or antagonistic function influenced by the subcellular localization [37,38]. ATRA exerts its transcriptional actions by binding to nuclear receptors. Since Akt activation is independent of transcriptional mechanisms and RARα is the major mediator of transcription-independent ATRA effects [30], we explored the possible association between RARα and Akt. Our results show that RARα interacted with and activated Akt in response to ATRA treatment, which is consistent with the finding that over-expression of RARα increases Akt phosphorylation in COS-7 cells [11]. In addition, RARα is recruited to the plasma membrane, where it became co-localized with Akt in response to ATRA treatment. These results suggest that ATRA promotes the formation of a signaling complex at the plasma membrane in a RARα-dependent manner. Consistent with these data, a pool of RARα is located in lipid rafts forming complexes with signaling proteins as Gαq in response to retinoic acid [39]. RARα has been shown to interact with PI3k at the plasma membrane [11]. The formation of this signaling complex at the plasma membrane regulates Rac activation through the PI3k/Akt pathway to promote cellular invasion, a result that is consistent with the finding that ATRA promotes activation of Rac in neuroblastoma cells [40] and increases the invasion of pancreatic cancer cells [7,41] and promotes MMP-9 expression through RARα [42]. In addition, we evaluated the effect of ATRA treatment on apoptosis. The results showed that ATRA exerts a protective effect against apoptosis. However, PI3k/Akt pathway inhibition promoted apoptosis via activation of caspase-3. Studies in acute promyelocytic leukemia cells have shown that treatment with the PI3k inhibitor reverses the protective effect of ATRA against apoptosis [43]. Additionally, recent reports have shown that Akt activation suppresses the transactivation of RARα in lung cancer cells [44]. This suggests that Akt negatively modulates the transcriptional actions of ATRA by inhibiting the expression of tumor suppressor genes such as RARβ2 and p53. To address this issue, we evaluated the expression of RARβ2, one of the target genes of ATRA. Our results showed that the over-expression of an active form of Akt (Myr-Akt) blocks the expression of RARβ2, whereas the inactive form of Akt (Akt-K179M) or PI3k inhibitor treatment increases the expression of RARβ2. In addition, over-expression of Myr-Akt substantially reduces p53 expression, other target gene of ATRA [28,45], whereas treatment with proteasome inhibitor (MG132) not restores p53 expression, indicating that Akt regulates p53 expression to transcriptional level. Consistent with these results, the PI3k/Akt pathway induces the down-regulation of RARβ2 mRNA and protein levels [27,46]. Finally, we tested the role of the PI3k/Akt pathway in cell proliferation. The results showed that treatment with PI3k inhibitor (15e) exerts a modest anti-proliferative effect. These results indicate that another kinase, such as ERK, regulates proliferation in lung cancer cells.

**Figure 8:**
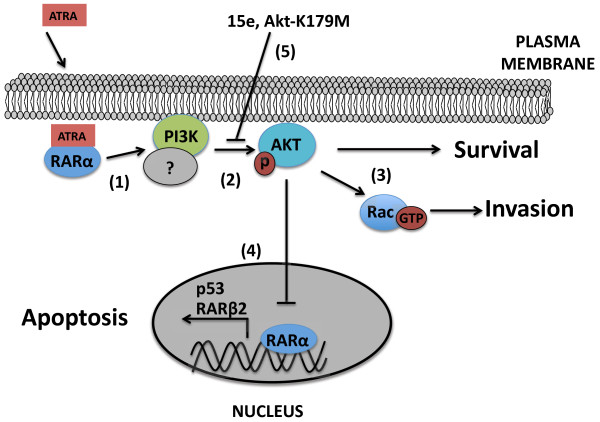
**Model depicting the molecular mechanism of ATRA resistance in lung cancer.** ATRA promotes RARα recruitment to the membrane, where it activates the PI3k-Akt pathway (1–2). Akt activation promotes cellular survival and cellular invasion (3). Akt represses RARβ2 and p53 expression (4). PI3k-Akt inhibition restores sensitivity to ATRA treatment and blocks survival and invasion (5).

Taken together, our results suggest that targeting the PI3k-Akt signaling pathway is a potential therapeutic strategy against ATRA-resistance in lung cancer. Follow-up experiments, such as proteomic analyses using mass spectrometry to identify scaffold proteins that regulate the complex assembly of the PI3k-Akt pathway, will be worthwhile for improving our understanding of this proposed mechanism. In agreement with this proposal, recent reports show that cellular retinol–binding protein-I (CRBP-I) decreases the heterodimerization of the catalytic subunit of PI3k with its regulatory subunit in transformed breast cell lines [47]. Based on the results in this study, we propose a model depicting the mechanism of ATRA resistance in lung cancer, as shown in Figure 8. In our model, ATRA binds to RARα to promote its localization at the plasma membrane (step 1). RARα subsequently promotes the recruitment and activation of the PI3k-Akt pathway. The formation of this signaling complex suggests the involvement of scaffold proteins in its assembly (step 2). Akt activation promotes cellular survival and cellular invasion through Rac-GTPase (step 3). Akt suppresses the transactivation of RARα and decreases the expression of RARβ2 (step 4). PI3k-Akt inhibition with 15e or over-expression of an inactive form of Akt (K179M) blocks survival and invasion, restoring the expression of tumor suppressors RARβ2 and p53 (step 5).

## Conclusions

In this study, we provide information on new molecular mechanisms by which lung cancer cells become resistant to ATRA treatment. Our results demonstrate that ATRA promotes PI3k-Akt activation via transcription-independent mechanisms mediated by the RARα-Akt interaction. PI3k-Akt activation by ATRA promotes invasion through Rac-GTPase activation and cell survival, whereas treatment combining ATRA and a PI3k inhibitor or over-expression of an inactive form of Akt suppresses invasion and cell survival, increasing the levels of active caspase-3 and the tumor suppressor RARβ2. In conclusion, activation of Akt blocks the transcriptional effects of ATRA, promotes invasion and cell survival, and confers resistance to retinoic acid treatment in lung cancer cells. These findings provide strategies for the design of drugs that combine ATRA and PI3k inhibitors for lung cancer treatment, a treatment modality that should be clinically evaluated.

## Materials and methods

### Cell lines and treatments

A549 cells were routinely grown in DMEM/F12 medium supplemented with 10% fetal bovine serum (FBS), 100 IU/ml penicillin, 100 μg/ml streptomycin at 37°C in a 5% CO_2_ atmosphere. All-*trans* retinoic acid (ATRA) was purchased from Sigma-Aldrich. The PI3k kinase inhibitor, 15e (3-[4-(4-morpholinyl) thieno [3,2-d]pyrimidin-2-yl]-phenol), was purchased from Enzo Life Science and the pan-retinoic acid receptor inverse agonist BMS 493 (4-[(1E)-2-[5,6-dihydro-5,5-dimethyl-8-(2-phenylethynyl)-2-naphthalenyl]ethenyl]benzoic acid), was purchased from Tocris Bioscience. The proteasome inhibitor MG132, was purchased from Sigma-Aldrich. The different compounds were dissolved in dimethylsulfoxide and added to the culture medium at the indicated concentrations.

### Western blot and immunoprecipitation

Whole-cell extracts were obtained by lysis of A549 cells in lysis buffer [20 mM Tris–HCl (pH 7.5), 1 mM EDTA, 150 mM NaCl, 1% Triton X-100, 1 mM sodium vanadate, 1 mM NaF, 10 mM β-glycerophosphate, 1 mM phenylmethylsulfonyl fluoride, and 1.2 mg/ml complete protease inhibitor cocktail; Roche]. The protein extracts were forced through a 22-gauge needle 10 times and centrifuged for 10 min at 14,000 rpm at 4°C and protein concentration was determined by the bicinchoninic acid BCA Protein Assay (Pierce). Approximately 25 μg of protein were separated on 10% SDS-PAGE and transferred to PVDF membranes and then incubated with primary antibodies: anti-phospho-Akt (sc-7985-R; Santa Cruz), anti-Akt (P-2482; Sigma-Aldrich), anti-p53 (sc-126; Santa Cruz) and anti-actin (sc-1616; Santa Cruz). Immunodetection was performed using a fluorescent substrate system (Millipore). Densitometry analysis of western blots was performed using the public domain NIH ImageJ software.

The interactions between endogenous RARα receptors and Akt was assessed in A549 cells that were serum-starved for 18 h and stimulated with 5 μM ATRA, as indicated in the figures. Confluent cultures were washed with PBS, followed by lysis at 4°C. The protein extracts were forced through a 22-gauge needle 10 times and centrifuged for 10 min at 14,000 rpm at 4°C. The supernatants were incubated for 12 h at 4°C with 5 μg/ml anti-RARα (MCA4135Z; Serotec). The immune complexes were recovered by incubation for 2 h at 4°C with protein G-sepharose (25 μl, 10–1241; Invitrogen). Beads were washed three times with lysis buffer and boiled in 1× Laemmli sample buffer. Immunoprecipitated proteins were fractionated on 10% SDS-PAGE and transferred to a PVDF membrane (Millipore). Expression of proteins and putative interactions were detected by western blot using an anti-Akt antibody (P-2482; Sigma-Aldrich). The mouse monoclonal anti-rabbit IgG, light chain specific antibody (211-032-171; Jackson Immuno Research) was used to detect primary antibody.

### Immunofluorescence

A549 cells were grown on coverslips precoated with poly-L-lysine and the cells were serum-starved for 18 h and stimulated with 5 μM ATRA for the indicated times. Then, cells were fixed with 4% paraformaldehyde in PBS for 20 min at room temperature, washed three times with PBS, permeabilized with methanol for 6 min at -20°C and blocked with 1% BSA in PBS for 30 min. The cells were then incubated with the primary antibodies. In some experiments, cells were incubated with anti-RARα (MCA4135Z; Serotec) and anti-Akt (P-2482; Sigma-Aldrich) or anti-cleaved caspase-3 (sc-22171; Santa Cruz) followed by incubation with anti-mouse Alexa Fluor 532, anti-mouse Alexa fluor 647 or anti-goat FITC (sc-2024; Santa Cruz), respectively. The cells on coverslips were mounted on glass slides using Vectashield (Vector Laboratories). To visualize the subcellular distribution of RARα and Akt, the images were acquired with a FV1000 confocal laser-scanning microscope (Olympus) using a 63× objective, and for caspase-3 activation, the images were acquired with an Axiovert 40 CFL fluorescence microscope (Carl Zeiss) using a 100× objective.

### Rac activation assay

Activation of Rac-GTPase was assessed using the Rac activation assay kit (Millipore) according to the manufacturer’s indications. Briefly, cells were preincubated with 5 μM of 15e for 1 h and stimulated with 5 μM of ATRA, as indicated in the figure legends. Cell lysates were incubated with p21-activated kinase (PAK) binding domain-tagged agarose (10 μg) at 4°C for 2 h. The agarose beads were washed three times with lysis buffer (Millipore) supplemented with phosphatase inhibitors and boiled for 5 min in 1× Laemmli sample buffer. Activated Rac was detected by western blot with Rac antibody (Millipore).

### Transfection

For transient transfection, cells were transfected using Lipofectamine™ LTX plus reagent (Invitrogen) according to the manufacturer’s indications. The total amount of DNA in transfections was 4 μg/plate; the assay was performed 48 h after transfection. Expression of transfected constructs was determined by western blot using anti-HA monoclonal antibodies (Covance) and anti-GFP (MMS-118R; Covance). DNA constructs pcDNA3-Myr-HA-Akt, pEGFPC1-human APPL1 and pCMV5-HA-Akt-DN (K179M) were obtained from Addgene, a non-profit plasmid repository (http://www.addgene.org/).

### Invasion assay

Cell invasion was carried out using QCM 24-Well Cell Invasion Assay (Millipore) according to the manufacturer’s instructions. Briefly, the extracellular matrix of the insert (8 μm pore size) was rehydrated with serum-free medium, which was subsequently replaced with 250 μl of prepared serum-free suspension of cells transfected with empty vector, Myr-Akt or Akt K179M (1.0 × 10^6^ cells/ml). Then, 500 μl of medium containing 5 μM of ATRA was added to the lower chamber of the insert. Cells were incubated at 37°C in a 5% CO_2_ atmosphere for 24 h. Finally, cells were dissociated from the membrane according to the manufacturer’s instructions and then detected with CyQuant GR Fluorescent Dye. Fluorescence was measured at 480/520 nm in a Tecan Infinite M1000 plate reader.

### TUNEL assay

Detection of apoptosis was performed using the DeadEnd colorimetric TUNEL assay kit (Promega) according to the manufacturer’s instructions. Briefly, A549 cells were grown on coverslips precoated with poly-L-lysine and treated for 48 h with 5 μM of ATRA with or without 5 μM of 15e. After treatment, the cells were fixed with 4% paraformaldehyde in PBS and permeabilized with 0.2% Triton X-100 in PBS. Cells were incubated with recombinant terminal deoxynucleotidyl transferase (rTdT) and biotinylated nucleotides. Endogenous peroxidases were blocked with 0.3% hydrogen peroxide in PBS. The cells were incubated with Streptavidin-HRP, which binds to biotinylated nucleotides incorporated at the 3′-OH DNA ends present in apoptotic cells. Streptavidin-HRP labeled cells were detected by hydrogen peroxide and diaminobenzidine (DAB).

### Proliferation assay

A549 cells were seeded in a 96-well plate at a concentration of 10,000 cells/well in 100 μl of DMEM/F12. The cells were treated for 24 h with 5 μM of ATRA with or without 5 μM of 15e. Cell proliferation was measured using the 5-bromo-2′-deoxyuridine (BrdU) enzyme-linked immunosorbent assay (Roche) according to the manufacturer’s instructions. For the last 6 h of the 24 h treatment period, the cells were pulsed with BrdU. Absorbance at 370 and 492 nm was measured in a Tecan Infinite M1000 plate reader.

### Statistical analysis

Statistical significances of the differences among data were determined by analysis of variance and Newman-Keuls test or *t* test, when appropriate, using GraphPad Prism 5.0 software. *P* < 0.05 was considered as statistically significant. Values are presented as means ± SEM.

## Abbreviations

ATRA: All-*trans* retinoic acid; RARs: Retinoic acid receptors; TUNEL: Terminal deoxynucleotidyl transferase dUTP nick end labeling; NSCLC: Non-small cell lung cancer.

## Competing interests

The authors declare that they have no competing interests.

## Authors’ contributions

Conception and design: C H G-D R, A G-R. Financial support: C H G-D R. Collection and assembly of data: A G-R. Data analysis and interpretation: C H G-D R, A G-R. Manuscript writing: A G-R, M V, A G-C, E A-O, C H G-D R. Final approval of manuscript: A G-R, M V, A G-C, E A-O, C H G-D R. All authors read and approved the final manuscript.

## Supplementary Material

Additional file 1: Figure S1 ATRA activates the Akt pathway in H1944 and NL-20 cells. (A) Left, H1944 cells were serum-starved for 18 h and treated or non-treated (NT) with 5 μM of ATRA for the times indicated. Right, NL20 cells were serum-starved for 18 h and treated or non-treated (NT) with 5 μM of ATRA for the times indicated and total extracts were prepared. The phosphorylated form of Akt and total proteins levels were detected by western blot using specific antibodies. Click here for file

Additional file 2: Figure S2 Inhibition of the PI3k/Akt pathway increased RARβ2 expression. A549 cells were serum-starved for 18 h and preincubated for 1 h with 5 μM of 15e before ATRA treatment. The cells were subsequently treated or non-treated with 5 μM of ATRA for 48 h, total extracts were prepared and levels of protein were detected by western blot. Click here for file
