# Supplementary figures and images for: Activation of Akt pathway by transcription-independent mechanisms of retinoic acid promotes survival and invasion in lung cancer cells
Source: Mol Cancer. 2013 May 21;12:44. doi: 10.1186/1476-4598-12-44 (PMC3665688; doi:10.1186/1476-4598-12-44)

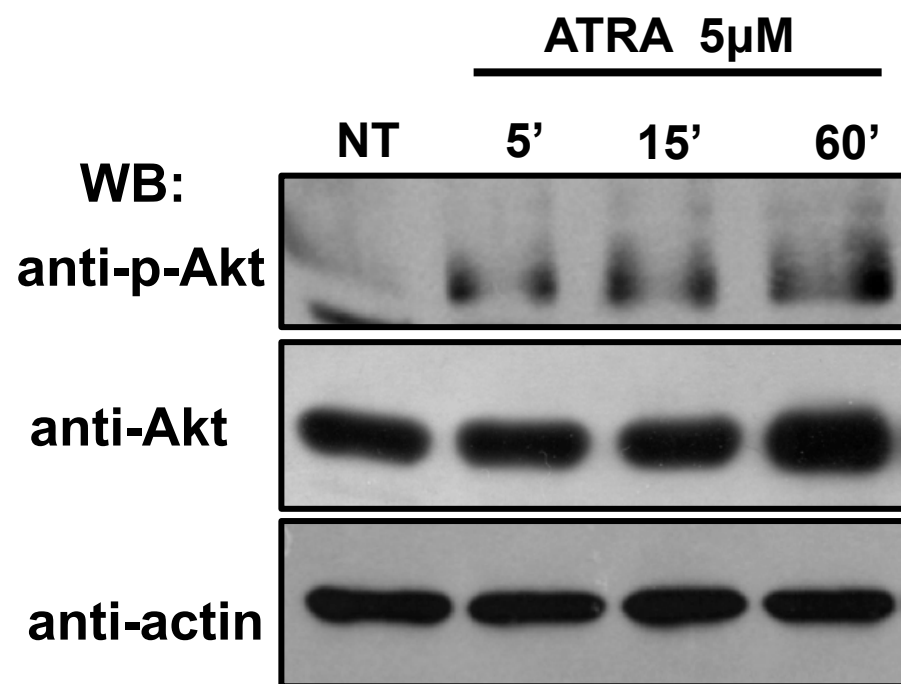

H1944

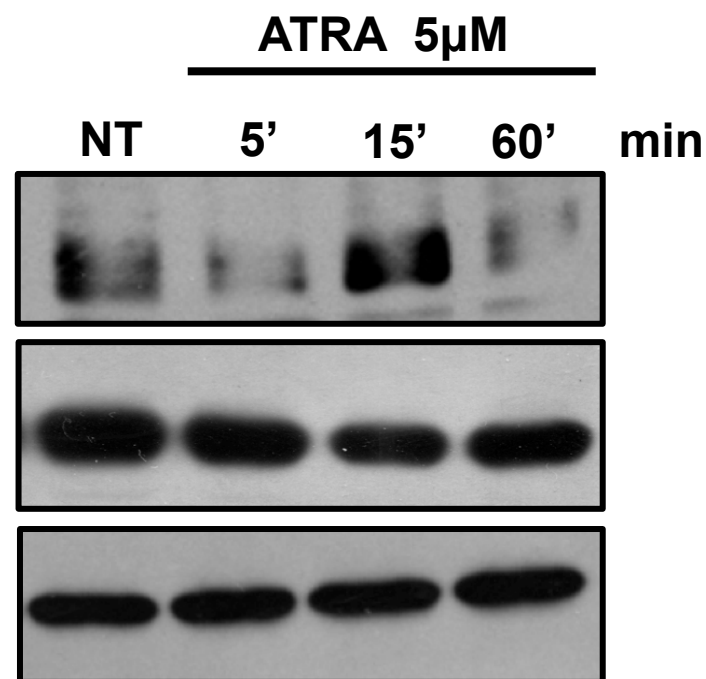

NL-20

Figure S1

Supplement: Additional file 1: Figure S1 — ATRA activates the Akt pathway in H1944 and NL-20 cells. (A) Left, H1944 cells were serum-starved for 18 h and treated or non-treated (NT) with 5 μM of ATRA for the times indicated. Right, NL20 cells were serum-starved for 18 h and treated or non-treated (NT) with 5 μM of ATRA for the times indicated and total extracts were prepared. The phosphorylated form of Akt and total proteins levels were detected by western blot using specific antibodies. [file 1476-4598-12-44-S1.pdf]

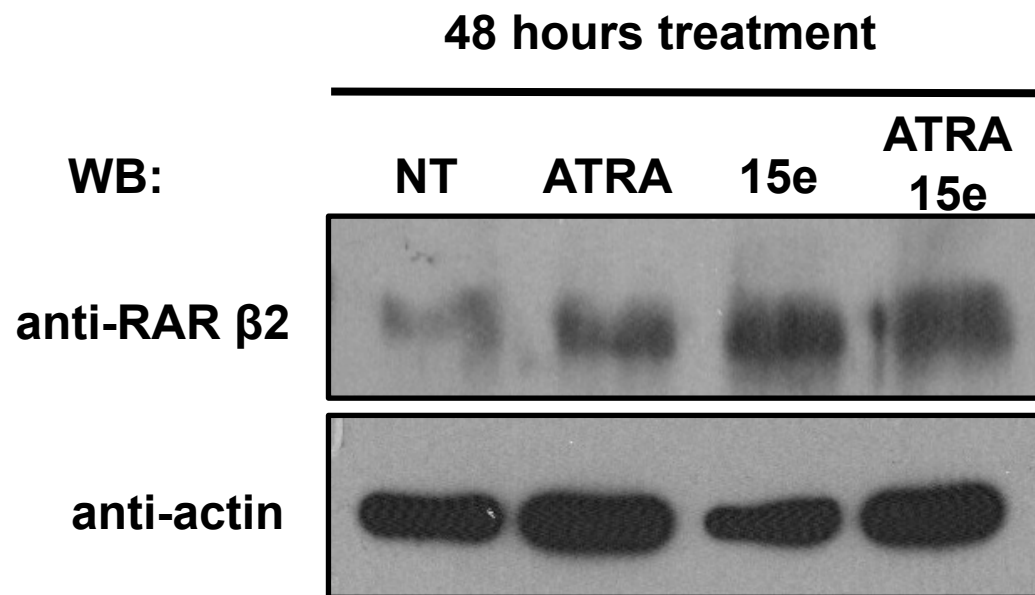

**Figure S2**

Supplement: Additional file 2: Figure S2 — Inhibition of the PI3k/Akt pathway increased RARβ2 expression. A549 cells were serum-starved for 18 h and preincubated for 1 h with 5 μM of 15e before ATRA treatment. The cells were subsequently treated or non-treated with 5 μM of ATRA for 48 h, total extracts were prepared and levels of protein were detected by western blot. [file 1476-4598-12-44-S2.pdf]
